# The utility of early gestational OGTT and biomarkers for the development of gestational diabetes mellitus: an international prospective multicentre cohort study

**DOI:** 10.1007/s00125-025-06517-0

**Published:** 2025-08-16

**Authors:** Evelyn A. Huhn, Grammata Kotzaeridi, Thorsten Fischer, Monja Todesco Bernasconi, Anne S. Richter, Mirjam Kunze, Eva Dölzlmüller, Heidi Jaksch-Bogensperger, Laura Weidinger, Daniel Eppel, Nicole Ochsenbein-Koelble, Elke Bäz, Bettina Winzeler, Andrea Tura, Helena Stach, Günther Schäfer, Shane V. van Breda, Lenka Vokálová, Irene Hoesli, Christian S. Göbl

**Affiliations:** 1 Department of Obstetrics and Gynaecology, University Hospital Basel, University Basel, Basel, Switzerland; 2 Clinic of Obstetrics and Prenatal Medicine, University Hospital Hamburg-Eppendorf, Hamburg, Germany; 3 Department of Obstetrics and Gynaecology, Division of Obstetrics and Feto-maternal Medicine, Medical University of Vienna, Vienna, Austria; 4 Department of Obstetrics and Gynaecology, Salzburger Landeskrankenhaus, Paracelsus Medical University, Salzburg, Austria; 5 Department of Obstetrics and Gynaecology, Cantonal Hospital Aarau, Aarau, Switzerland; 6 Department of Obstetrics and Gynaecology, University Hospital Zurich, Zurich, Switzerland; 7 Department of Obstetrics and Gynaecology, University Hospital Freiburg, Freiburg, Germany; 8 Department of Endocrinology and Diabetology, University Hospital Basel, Basel, Switzerland; 9 CNR, Institute of Neuroscience, Padova, Italy; 10 Department for Biomedical Research, Inselspital, Bern University Hospital, University of Bern, Bern, Switzerland

**Keywords:** Biomarkers, Early pregnancy, First-trimester screening, Gestational diabetes mellitus, OGTT, Risk stratification

## Abstract

**Aims/hypothesis:**

There is no clear consensus regarding accurate risk stratification in early pregnancy for later developing gestational diabetes mellitus (GDM). Therefore, this study aims to evaluate the predictive performance of an OGTT and several biomarkers in the first trimester of pregnancy. Their association with insulin action, beta cell function and requirement for insulin were additionally assessed.

**Methods:**

In this prospective cohort study, we included 657 pregnant women in six Central European centres. Patient history and anthropometric data were obtained, a blinded 75 g OGTT was performed and biochemical markers were assessed at a median gestational age of 13.4 weeks (IQR 12.7–14.1). Another OGTT was performed in later pregnancy to identify women with GDM. A detailed investigation of glucose homeostasis was performed at both visits in a subgroup of women.

**Results:**

Eighty-three women (12.6%) developed GDM. Progression to GDM was fairly well predicted by glucose concentrations during the early OGTT in terms of areas under the receiver operating characteristic curves (OGTT glucose at fasting: 0.68; OGTT glucose at 60 min: 0.74; OGTT glucose at 120 min: 0.72). Some biomarkers showed significant but modest predictive accuracy. Early gestational OGTT glucose concentrations were further associated with impaired insulin sensitivity and beta cell dysfunction, as well as the requirement for insulin in later pregnancy.

**Conclusions/interpretation:**

Although the accurate diagnosis of GDM before 24 weeks remains an ongoing discussion, dynamically assessed glucose concentrations during an early OGTT were closely associated with impaired glucose homeostasis and showed good predictive accuracy for later development of GDM as well as the requirement for insulin. These findings may be used to develop a protocol to distinguish between low- and high-risk mothers.

*Trial registration* ClinicalTrials.gov NCT02035059

**Graphical Abstract:**

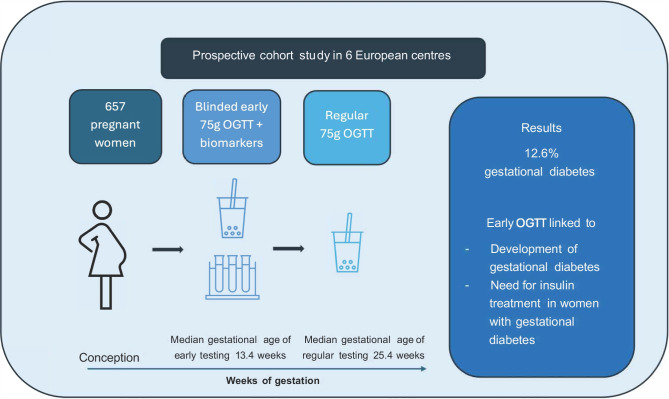

**Supplementary Information:**

The online version of this article (10.1007/s00125-025-06517-0) contains peer-reviewed but unedited supplementary material.


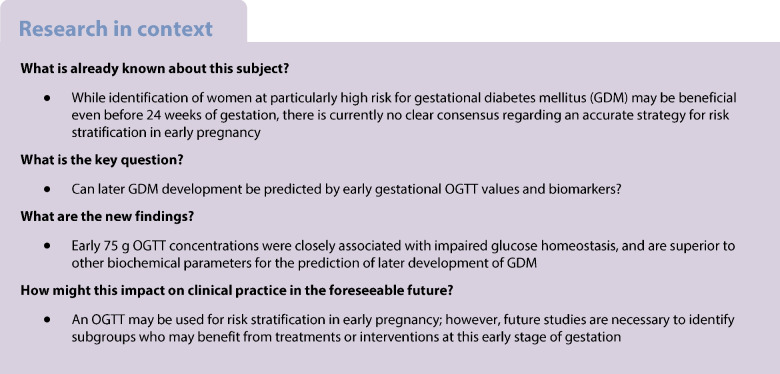


## Introduction

International guidelines recommend testing for gestational diabetes mellitus (GDM) in the late second trimester by use of a 75 g OGTT [1, 2]. While the identification of women with particularly high risk may be beneficial even before 24 weeks of gestation, there is currently no clear consensus on an accurate strategy for risk stratification in early pregnancy [3, 4]. In 2010, the International Association of Diabetes in Pregnancy Study Groups (IADPSG) consensus panel recommended that ‘early’ GDM should be recognised if fasting glucose is equal to or above 5.1 mmol/l (92 mg/dl) [1]. Later, some investigators claimed that there is insufficient data ‘to confidently recommend’ glucose thresholds for GDM diagnosis in the early gestational period [5]. Since an international multicentre study showed modest advantages of treating high-risk women with abnormal OGTT in early pregnancy [6], various healthcare organisations have updated their screening guidelines based on the latest evidence, although the specific recommendations sometimes differ [7, 8]. While the definition of ‘early’ GDM and the criteria for screening remain the subject of ongoing debate, glucose levels (fasting or during OGTT) in early pregnancy have been shown to be associated with impaired glucose metabolism [9, 10] and are therefore regarded as a risk factor for the later development of GDM [3, 11] as well as other adverse clinical outcomes [12]. Likewise, a number of routine biochemical markers, such as serum lipids, as well as other biomarkers (e.g. adipokines, lipocalins, placental hormones or glycated serum proteins), have been found to be associated with impaired glucose metabolism, and some of them showed promising predictive accuracy for later development of GDM, independently of or in addition to OGTT glucose concentrations [3, 13–19]. However, the prognostic accuracy of early OGTT testing, in combination with promising biomarkers, has been less well investigated.

Therefore, the present study aimed to evaluate and compare the predictive performance of a 75 g OGTT and several biochemical markers at the start of pregnancy for GDM progression. The associations with parameters of glucose homeostasis, such as insulin sensitivity and beta cell function, as well as with the requirement for insulin, were also determined.

## Methods

### Study design and participants

**Fig. 1:**
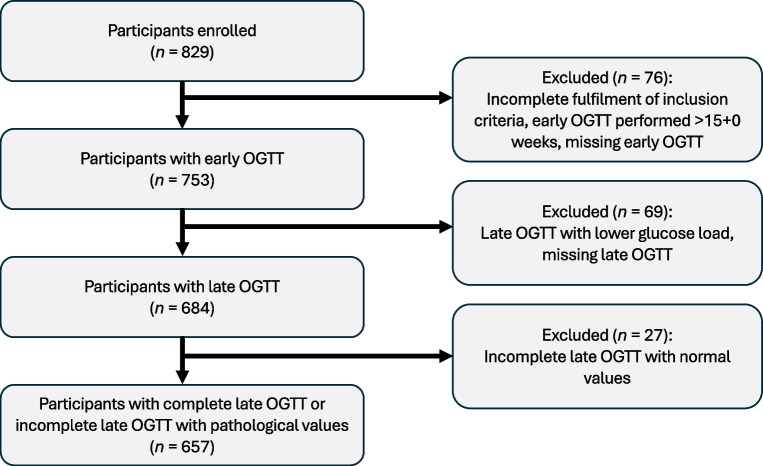
Flow chart of enrolments and exclusions

This prospective cohort study was conducted at six centres in Switzerland, Germany and Austria, and therefore represents a Central European population. The study protocol has been previously reported elsewhere [20]. The main study was registered under www.ClinicalTrials.gov (NCT02035059), and comprised 829 women who were recruited when attending the six Obstetrics and Prenatal Medicine outpatient departments for first-trimester screening. The recruitment period started in 2014 in Basel and in 2016 for the other study centres, and lasted until 2019. After excluding 172 women for various reasons (Fig. 1), 657 pregnant women remained (Aarau: *n*=45; Basel: *n*=270; Zurich: *n*=45; Vienna: *n*=198; Salzburg: *n*=39; Freiburg, *n*=60). Exclusion criteria were pre-existing diabetes, chronic infectious diseases (such as hepatitis or HIV infection), chronic kidney, liver or heart disease, or history of bariatric surgery or treatment with glucose-lowering medications (such as metformin) at study entry. Patients with fetal genetic, chromosomal or morphological abnormalities that required further clarification were not included. In contrast to a previously published secondary analysis of the same study, which was conducted on 636 women [21], in the current analysis we also included 13 patients with incomplete baseline OGTT data, as well as eight patients with incomplete late OGTT results but with pathological values, to maximise data utilisation. In the majority of the latter patients, the OGTT was discontinued after a diagnosis of GDM was established based on fasting glucose levels. The ‘early’ 2 h 75 g OGTT was performed at a median gestational age of 13.4 weeks (IQR 12.7–14.1); participants and doctors were blinded to the test results. Unblinding was performed if fasting or 2 h post-load glucose levels were ≥7.0 mmol/l (126 mg/dl) or 11.1 mmol/l (200 mg/dl), respectively. These patients were excluded from the study and appropriately treated thereafter. Pre-gestational BMI, ethnicity, maternal age, obstetric history and parity, as well as family history of diabetes and history of GDM in previous pregnancy, were additionally assessed. We performed a laboratory examination on fasting samples at the baseline visit to assess the biochemical markers insulin, triglycerides/triacylglycerols, HDL-cholesterol, adiponectin, lipocalin-2, placental growth factor (PlGF) and vitamin D, as well as glycated serum proteins (glycated fibronectin, glycated albumin and fructosamine). A second ‘diagnostic’ 2 h 75 g OGTT was performed at a follow-up visit between 24 and 28 weeks of gestation, whereby the patients were classified as having GDM if the fasting, 60 min or 120 min glucose concentration was ≥5.1 mmol/l (92 mg/dl), ≥10.0 mmol/l (180 mg/dl) or ≥8.5 mmol/l (153 mg/dl), respectively. However, in 74 instances, the second examination was performed outside this time range. The median time for OGTT testing was 25.4 weeks (IQR 24.6–26.1). All women diagnosed with GDM received medical nutrition therapy and lifestyle advice for 30 min, and were advised on capillary blood glucose monitoring. In line with local and international guidelines, insulin was indicated if either fasting or 1 h postprandial glucose exceeded 5.3 mmol/l (95 mg/dl) or 7.8 mmol/l (140 mg/dl), respectively, for more than two measures in a period of 1 week [22, 23]. The study participants were followed-up until delivery to assess pregnancy outcomes, such as birthweight and Caesarean section rate. Birthweight percentiles were calculated based on international standards [24]. Large for gestational age (LGA) and small for gestational age (SGA) were defined as birthweights above or below the 90th and 10th percentiles, respectively.

### Laboratory analyses

For the OGTT, plasma glucose was measured by the automated colorimetric–enzymatic method (hexokinase/glucose 6-phosphate-dehydrogenase). For a more detailed investigation of glucose metabolism, HbA_1c_ as well as insulin and C-peptide during the OGTT (at fasting and at 60 and 120 min after the oral glucose load) were assessed in a subgroup of participants (only women from Vienna) at both visits. Insulin (CV 4–7%) and C-peptide (CV 3–4%) were measured by chemiluminescence immunoassays. HbA_1c_ was assessed by HPLC (IFCC-standardised and DCCT-aligned), with a CV of 1.8%.

For measurement of biochemical markers, additional serum and plasma were taken at the fasting stage of the 75 g OGTT, spun at 3000 *g* at 7 min, aliquoted and stored at −80°C. Measurements of triglycerides/triacylglycerols, HDL-cholesterol, adiponectin, lipocalin-2, PlGF, 25-hydroxyvitamin D_3_ (vitamin D), glycated albumin, glycated fibronectin and fructosamine were performed on the banked maternal serum samples. The samples were thawed for analysis of the listed biomarkers. Triglycerides/triacylglycerols and HDL-cholesterol, fructosamine and PlGF were measured using a Roche Cobas C8000 chemistry analyser (Roche Diagnostics, Mannheim, Germany). The Roche Elecsys assay was used for the analysis of PlGF. Adiponectin, lipocalin-2, vitamin D, glycated albumin and glycated fibronectin were measured using time-resolved fluorescence immunoassays on an automated platform (AutoDELFIA immunoanalyser; PerkinElmer, Turku, Finland). The adiponectin assay was performed using a RayBio human adiponectin ELISA kit (RayBio, Norcross, GA, USA) with a monoclonal capture antibody and detection using a biotinylated antibody and HRP-conjugated streptavidin. The adiponectin assay range was 25–18,000 pg/ml, with an intra-assay CV <10%. Lipocalin-2 was measured using a RayBio human lipocalin-2 ELISA kit (RayBio). For monoclonal capture antibody detection, a biotinylated antibody was added, as well as HRP-conjugated streptavidin. The lipocalin-2 assay range was 4–1000 pg/ml, with an intra-assay CV <10%. Vitamin D was analysed using a monoclonal antibody 25-hydroxy vitamin D ELISA kit (DIAsource ImmunoAssays, Louvain-la Neuve, Belgium). The range was 4.4–133 ng/ml, with CV <10%. Glycated albumin was measured using a human glycated albumin ELISA kit (Abbexa, Cambridge, UK). The test range was 18.75–1200 pmol/ml, with CV <10%. Glycated fibronectin was analysed using a Lumella glycated fibronectin ELISA kit (AL-160-OEM) (DiabetOmics, Beaverton, OR, USA) using a monoclonal capture antibody and a biotinylated *Sambucus nigra* antibody. Labelling was performed using high-sensitivity HRP-conjugated streptavidin. The test range was 50–800 μg/ml, with CV <10%. The laboratory personnel were blinded to the GDM status, maternal characteristics or clinical outcomes of the participating women.

### Calculations of glucose homeostasis

Whole-body insulin sensitivity was assessed using the composite index (ISI-comp) [25]. Insulin secretion was examined by an insulinogenic index calculated as AUC-I/AUC-G at 0–120 min, where AUC-I and AUC-G represent the AUC for insulin and glucose, respectively [26]. The extent to which the pancreatic beta cells can adapt to impaired insulin action was assessed by the insulin secretion–sensitivity index 2 (ISSI-2), calculated as the product of ISI-comp and AUC-I/AUC-G at 0–120 min [27].

### Ethics statement

The study was approved by the institutional review boards of the participating centres: Basel (EKNZ Basec-No. PB_ 2016–00611, for Basel, Aarau and Zurich); Freiburg (EK-No. 127/16); Salzburg (EK-No. 415-E/2014/11–2016); and Vienna (EK-No. 1079/2016). The study was performed in accordance with the Declaration of Helsinki, and written informed consent was obtained from all participants.

### Statistical analysis

Continuous variables are summarised using means ± SD for normally distributed variables or median and IQR for skewed distributions, and were compared using Welch’s *t* test or the Wilcoxon rank-sum test as appropriate. Categorical variables are summarised as counts and percentages and were compared using Pearson’s χ^2^ test. Associations between continuous variables were examined using Spearman’s rank correlation (ρ).

Associations between binary outcomes (e.g. GDM or LGA status) and explanatory variables (such as OGTT glucose and biomarkers) were assessed using binary logistic regression. The 95% CI for the OR were calculated using the likelihood ratio statistic, whereby the OR represents the relative change in the odds of developing a specific disorder (such as GDM or GDM with requirement for insulin) per 1 unit change of the respective predictor variable. Discrimination, defined as the ability of a predictor to separate pregnant women with a disease from those without the disease, was assessed using area under the receiver operating characteristic curves (AUROC).

Recursive partitioning was used to derive measures of variable importance, which were calculated as the mean difference in predictive accuracy before and after random permutation of the values of a predictor variable over all (i.e. 10^5^) random decision trees [28]. As a further approach we used L1-regularised logistic regression (sometimes called the least absolute shrinkage and selection operator, LASSO) to evaluate the effect of multiple predictors on a binary outcome (such as GDM status) and to select the most important predictor. Thereby, the shrinkage boundary was set to the largest value of L1 such that the cross-validated error is within one SE of the minimum [29]. The random forest method and L1-regularised regression were performed on complete cases, i.e. excluding those with missing values for main explanatory variables. This subgroup comprised 545 women (66 of whom developed GDM, of whom 21 received insulin).

The main analysis used the WHO 2013/IADPSG criteria [1, 2], but other cut-offs were also tested in sensitivity analyses. The first sensitivity analysis was performed after excluding 22 women who met alternative GDM diagnosis criteria in early pregnancy (fasting glucose ≥5.3 mmol/l and/or 1 h glucose ≥10.6 mmol/l and/or 2 h glucose ≥9.0 mmol/l [30]), and those who did not meet these criteria but had one or more missing glucose values during the early OGTT (*n*=14). Of the remaining 621 women, 68 developed GDM (17 of whom required insulin). In the second sensitivity analysis, we excluded 47 women who met the WHO diagnosis criteria (fasting glucose ≥5.1 mmol/l and/or 1 h glucose ≥10.0 mmol/l and/or 2 h glucose ≥8.5 mmol/l [1, 2]) and those with one or more missing glucose values during the early OGTT (*n*=14). Of the remaining 596 women, 55 developed GDM (13 of whom required insulin).

Statistical analysis was performed using R version 4.4.2, and contributed packages (especially ‘pROC’, ‘randomForest’ and ‘glmnet’, as well as ‘ggplot2’ and ‘corrplot’ for visualisations) [31]. A two-sided *p* value of ≤0.05 was considered statistically significant, and *p* values were interpreted in an explorative manner.

### Sample size justification

As previously reported, we estimated that 748 patients were required (given a GDM prevalence of 10.9% and a dropout rate of 15%) for a proposed AUC of 0.9 with a lower boundary of 0.8 (i.e. the lower limit of the 95% CI is >0.8) [20].

## Results

### Baseline characteristics of the study group

A total of 83 women (12.6%) developed GDM and 574 women remained normal glucose-tolerant (NGT). The prevalence of GDM was not significantly different between the centres (Aarau: 8.9%; Basel: 11.5%; Zurich: 20.0%; Vienna: 14.1%; Salzburg: 10.3%; Freiburg: 11.7%, *p*=0.583). A comparison of baseline characteristics, OGTT glucose concentrations and biomarkers is provided in Table 1. Mothers who developed GDM showed significantly higher pre-gestational BMI and higher glucose OGTT concentrations at the baseline visit, as well as elevated levels of fasting insulin, triglycerides/triacylglycerols, glycated fibronectin and lower adiponectin. Moreover, being of self-reported non-European descent as well as history of GDM in a previous pregnancy were more often observed in mothers with progression to GDM.

**Table 1:** Main characteristics of the study sample in early pregnancy

	*n*^a^	NGT	*n*^a^	GDM	*p* value
		(*n*=574)		(*n*=83)	
Age (years)	574	32.1±5.1	83	32.5±4.8	0.522
Parity ≥1	574	247 (43.0)	83	36 (43.4)	0.953
GDM in previous pregnancy	530	18 (3.4)	70	6 (8.6)	0.038
Ethnicity^b^	564	79 (14.0)	80	21 (26.3)	0.005
Family history of diabetes	556	165 (29.7)	79	32 (40.5)	0.052
Smoking status (current smokers)	536	45 (8.4)	75	6 (8.0)	0.908
Height (cm)	571	166±6.6	81	164±5.8	0.011
Weight before pregnancy (kg)	569	65.6±13.4	81	70.8±17.5	0.011
BMI before pregnancy (kg/m^2^)	568	23.8±4.6	81	26.2±6.1	<0.001
OGTT glucose levels (mmol/l)					
0 min	572	4.40±0.39	83	4.67±0.51	<0.001
60 min	569	5.97±1.71	82	7.61±1.89	<0.001
120 min	565	4.92±1.24	82	6.21±1.72	<0.001
Insulin (pmol/l)	524	48.6 (36.1–68.1)	72	58.7 (43.2–100.9)	0.001
Triglycerides/triacylglycerols (mmol/l)	535	1.28±0.44	74	1.42±0.49	0.023
HDL-cholesterol (mmol/l)	535	1.73±0.37	74	1.71±0.38	0.582
Adiponectin (µg/ml)	556	8.5 (5.7–13.1)	79	6.2 (5.1–10.5)	<0.001
Lipocalin-2 (ng/ml)	556	45.7 (23.9–91.1)	79	51.4 (24.7–104.3)	0.427
PlGF (ng/l)	535	49.5±22.1	74	52.1±22.1	0.355
Vitamin D (nmol/l)	556	48.7±24.0	79	48.9±32.2	0.953
Glycated albumin (µmol/l)	506	2.23 (1.39–3.40)	72	2.13 (1.18–3.07)	0.130
Glycated fibronectin (mg/dl)^c^	557	26.8 (21.1–33.7)	79	30.5 (24.6–36.0)	0.015
Fructosamine (µmol/l)	536	244±29	73	238±29	0.073

Data are means ± SD or median (IQR) for continuous variables and *n* (%) for categorical variables for women who remained NGT and those who developed GDM in mid-pregnancy

^a^The value of *n* varies based on the amount of missing data

^b^Number of participants who were not of European descent

^c^To convert mg/dl to μg/ml, multiply by 10

### Associations with parameters of glucose homeostasis

**Fig. 2:**
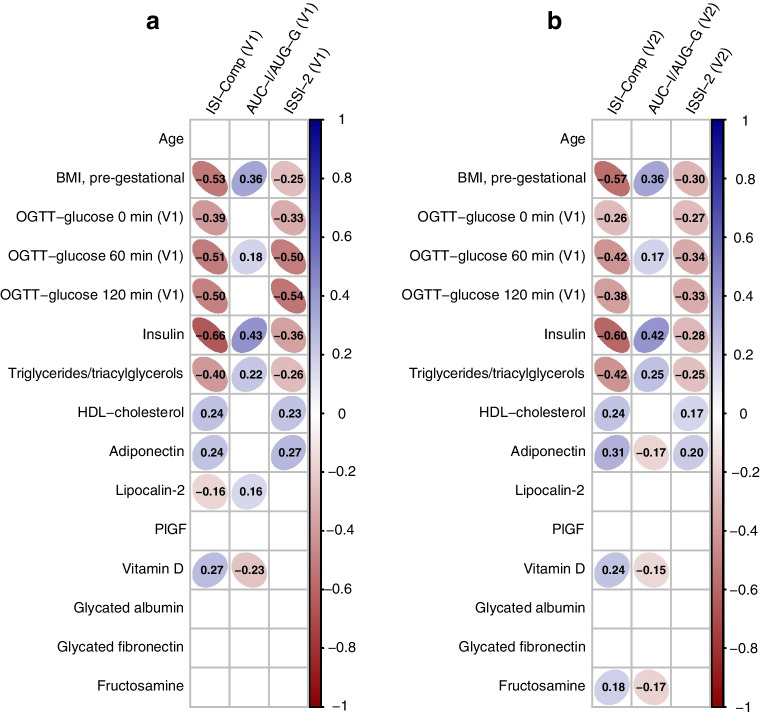
Correlation map representing the association of early gestational biomarkers with insulin resistance (ISI-Comp), insulin secretion (AUC-I/AUC-G) and beta cell function (ISSI-2) assessed in early pregnancy (baseline visit, **a**) and mid-gestation (follow-up visit, **b**). The magnitude of correlation is indicated by the shape of the ellipses (where a circle represents no correlation) and their colour (a darker colour represents higher correlation; positive = blue, negative = red). Numbers indicate the value of the Spearman coefficient (ρ). Blank cells indicate that the association is not significant

The associations of baseline parameters (OGTT glucose concentrations and biomarkers) with whole-body insulin sensitivity, insulin secretion and beta cell function are visualised in Fig. 2. Pre-gestational BMI, as well as fasting glucose, insulin and glucose concentrations during the baseline OGTT, were associated with impaired insulin action and decreased beta cell function at both visits. Glucose levels at fasting (ρ: 0.32, *p*<0.001) and during the OGTT (ρ: 0.27, *p*<0.001 and ρ: 0.25, *p*<0.001, for 60 and 120 min glucose levels, respectively) were further associated with higher HbA_1c_ at baseline. An inverse correlation was found between maternal triglycerides/triacylglycerols and insulin sensitivity and beta cell function. However, the effect of the remaining biomarkers was modest, although sometimes significant. The reported correlations were comparable between the baseline (Fig. 2a) and the follow-up visit (Fig. 2a).

### Prediction of GDM by ‘early’ OGTT glucose levels and biomarkers

**Fig. 3:**
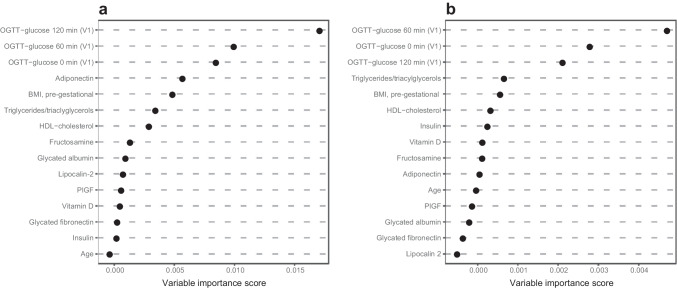
Variable importance scores for biomarkers assessed in early pregnancy for the prediction of GDM (**a**) and GDM with the requirement for insulin (**b**) in mid-pregnancy

Table 2 shows the univariable association of baseline parameters with progression to GDM. Pre-gestational BMI, fasting glucose and insulin, both post-load OGTT glucose concentrations, triglycerides/triacylglycerols and adiponectin predicted progression to GDM, and the highest AUROC values (ranging from 0.68 to 0.74) were observed for fasting and both post-load glucose values during the OGTT. Recursive partitioning indicated the highest variable importance scores for glucose concentration at fasting and both post-load glucose values (Fig. 3a). These predictors were also selected by L1-regularised regression, whereas all other predictors were excluded (i.e. were shrunken to zero). The L1-regularised combination of fasting and both OGTT glucose values predicted the later development of GDM with an AUROC value of 0.75 (95% CI 0.69–0.82).

**Table 2:** Univariable analysis for predictors of developing GDM in mid-pregnancy

Parameter	OR	95% CI	*p* value	AUROC	95% CI
Age (years)	1.01	0.97, 1.06	0.540	0.51	0.45, 0.58
BMIPG (kg/m^2^)	1.09	1.04, 1.13	<0.001	0.63	0.56, 0.70
OGTT glucose levels (mmol/l)					
0 min	5.06	2.83, 9.37	<0.001	0.68	0.61, 0.75
60 min	1.64	1.44, 1.89	<0.001	0.74	0.68, 0.79
120 min	1.92	1.62, 2.31	<0.001	0.72	0.65, 0.78
Insulin (pmol/l)^a^	1.90	1.29, 2.79	0.001	0.62	0.55, 0.69
Triglycerides/triacylglycerols (mmol/l)	1.87	1.13, 3.07	0.013	0.58	0.51, 0.66
HDL-cholesterol (mmol/l)	0.83	0.42, 1.59	0.569	0.51	0.44, 0.59
Adiponectin (µg/ml)^a^	0.55	0.39, 0.78	<0.001	0.62	0.55, 0.69
Lipocalin-2 (ng/ml)^a^	1.11	0.85, 1.46	0.443	0.53	0.46, 0.60
PlGF (ng/l)	1.01	0.99, 1.02	0.352	0.53	0.46, 0.60
Vitamin D (nmol/l)	1.00	0.99, 1.01	0.941	0.53	0.46, 0.60
Glycated albumin (µmol/l)^a^	0.72	0.52, 1.01	0.053	0.56	0.48, 0.63
Glycated fibronectin (µg/ml)^a^	1.48	0.80, 2.86	0.228	0.58	0.52, 0.65
Fructosamine (µmol/l)	0.99	0.98, 1.00	0.069	0.57	0.50, 0.64

^a^Logarithmic transformation (log_e_) was applied

BMIPG, pre-gestational BMI

### Prediction of GDM with requirement for insulin

A total of 25 GDM patients required insulin (indicating a more severe course of the disease). Table 3 shows the univariable association of baseline parameters with GDM and requirement for insulin. Pre-gestational BMI, fasting glucose and insulin, both post-load OGTT glucose concentrations, HDL-cholesterol and adiponectin predicted progression to GDM with requirement for insulin, and the highest AUROC values were observed for fasting and glucose concentrations during the OGTT (ranging from 0.71 to 0.79). Recursive partitioning indicated the highest variable importance scores for fasting and post-load glucose values (Fig. 3b). L1-regularised regression selected fasting and OGTT glucose at 60 min as predictors, whereas all other predictors were excluded from the model (i.e. were shrunken to zero). The L1-regularised combination of fasting and OGTT glucose at 60 min predicted the later development of GDM requiring insulin, with an AUROC value of 0.85 (95% CI 0.78–0.93).

**Table 3:** Univariable analysis for predictors of developing GDM in mid-pregnancy with requirement for insulin

Parameter	OR	95% CI	*p* value	AUROC	95% CI
Age (years)	1.01	0.94, 1.10	0.722	0.51	0.39, 0.64
BMIPG (kg/m^2^)	1.10	1.04, 1.17	<0.001	0.67	0.55, 0.79
OGTT glucose levels (mmol/l)					
0 min	8.94	3.71, 22.8	<0.001	0.77	0.66, 0.88
60 min	1.83	1.46, 2.34	<0.001	0.79	0.70, 0.88
120 min	1.69	1.29, 2.21	<0.001	0.71	0.60, 0.82
Insulin (pmol/l)^a^	2.29	1.25, 4.05	0.005	0.66	0.55, 0.78
Triglycerides/triacylglycerols (mmol/l)	2.12	0.93, 4.51	0.060	0.59	0.45, 0.73
HDL-cholesterol (mmol/l)	0.30	0.09, 0.96	0.049	0.59	0.47, 0.71
Adiponectin (µg/ml)^a^	0.48	0.28, 0.80	0.005	0.67	0.55, 0.78
Lipocalin-2 (ng/ml)^a^	1.16	0.74, 1.85	0.520	0.54	0.40, 0.68
PlGF (ng/l)	1.00	0.98, 1.01	0.691	0.51	0.40, 0.62
Vitamin D (nmol/l)	1.01	0.99, 1.02	0.068	0.51	0.39, 0.64
Glycated albumin (µmol/l)^a^	0.64	0.38, 1.09	0.095	0.60	0.48, 0.72
Glycated fibronectin (µg/ml)^a^	0.63	0.30, 1.59	0.267	0.46	0.34, 0.58
Fructosamine (µmol/l)	0.99	0.98, 1.01	0.411	0.51	0.40, 0.62

^a^Logarithmic transformation (log_e_) was applied

BMIPG, pre-gestational BMI

### Sensitivity analyses

A sensitivity analysis was performed after excluding women who met the alternative GDM diagnosis criteria in early pregnancy [30]. Compared with the entire cohort, variable importance metrics (as derived by random forest analysis) revealed lower importance scores for fasting glucose in the prediction of later GDM as well as of GDM with the requirement for insulin, whereas both post-load glucose values remained as the strongest predictors (electronic supplementary material ESM Fig. 1). In another sensitivity analysis, we excluded women who met the WHO diagnosis criteria and had missing values for the early OGTT. The OGTT glucose value at 60 min remained the strongest predictor in the random forest analysis (ESM Fig. 2).

### Pregnancy outcomes

Women who developed GDM in later pregnancy did not markedly differ from those who remained NGT with regard to pregnancy outcomes. Neonatal weight (3.4±0.5 vs 3.3±0.5 kg, *p*=0.150) and birthweight percentile (60.0±27.6 vs 55.6±28.0 percentiles, *p*=0.184), as well as the rates of LGA (15.0 vs 12.2%, *p*=0.502), SGA (5.0 vs 8.5%, *p*=0.188) and delivery by Caesarean section (32.0 vs 36.1%, *p*=0.449) were not statistically different between the groups (NGT vs GDM). Logistic regression identified a modest association of maternal pre-gestational BMI with LGA (*p*=0.032), but no significant associations were reported for early gestational glucose concentrations at fasting (*p*=0.862) or for either OGTT values (60 min: *p*=0.276; 120 min: *p*=0.057).

### Comment on sample size

Although some of the assumptions were not met (in particular, glucose concentrations and biomarkers showed less favourable predictive accuracy in terms of discrimination), an effective sample size of 657 women (including 83 GDM patients as observed in our study) can identify an AUROC value of at least 60.7%, compared with the non-informative AUROC value of 50%, with a power of 90% and a two-sided α-error probability of 5%.

## Discussion

This study aimed to evaluate the accuracy of glucose concentrations assessed during an ‘early’ OGTT, in addition to several biomarkers at the beginning of pregnancy in predicting the later development of GDM. We found that fasting and especially dynamically assessed glucose concentrations during the OGTT, as well as biochemical parameters, higher maternal triglycerides/triacylglycerols and insulin levels as well as lower adiponectin, were significantly associated with progression to GDM. Glucose concentrations (at fasting and during the OGTT) were further associated with impaired glucose homeostasis (i.e. impaired whole-body insulin action and decreased beta cell function) and the requirement for insulin. To our knowledge, this is the first study emphasising the role of blinded early OGTT glucose values, in addition to fasting biochemical markers, in predicting the later development of GDM, including a detailed assessment of glucose metabolism at the time of the early OGTT.

While screening for undiagnosed overt diabetes at start of pregnancy is recommended by international guidelines [2, 22], there is actually no consensus regarding an appropriate testing strategy for GDM in early gestation. In fact, the proposed IADPSG thresholds were originally developed for the late second and early third trimester, and are therefore not necessarily applicable for use before 24 weeks [3]. The IADPSG threshold for fasting glucose (5.1 mmol/l, 92 mg/dl) has been evaluated in previous studies, suggesting modest prognostic accuracy [32–34]. The applicability of the IADPSG thresholds for either OGTT glucose at 60 min (10.0 mmol/l, 180 mg/dl) or 120 min (8.5 mmol/l, 153 mg/dl) before 24 weeks of gestation is also under debate [3]. Interestingly, some previous studies suggested that fasting glucose tends to decrease with advanced gestational age, whereas post-load glucose values tend to increase, suggesting that different thresholds are necessary in early and late pregnancy [32, 35]. Most recently, the Treatment of Booking Gestational Diabetes Mellitus (TOBOGM) trial showed modest benefits in terms of adverse neonatal outcomes (24.9 vs 30.5%), but no improvements in pregnancy-related hypertension or lean body mass of the newborn, when high-risk women were treated before 20 weeks of gestation [6]. It should be mentioned that, in addition to those with overt diabetes, patients with moderate fasting hyperglycaemia (6.1–6.9 mmol/l) were excluded from the TOBOGM study, and greater benefits may be identified if these patients were included. Conversely, a secondary analysis of our study cohort (although it was not powered for this research question) revealed low sensitivity of the proposed WHO cut-off values when used in early pregnancy [21]. Another study, primarily focused on pregnancies in obese women, found that early screening before 20 weeks of gestation did not improve adverse perinatal outcomes. These outcomes included the rate of macrosomia (>4000 g), primary Caesarean section, hypertensive disorders of pregnancy, shoulder dystocia, hyperbilirubinaemia and neonatal hypoglycaemia (assessed within 48 h after birth) [36]. Consequently, there are still open questions regarding the effectiveness of GDM screening in early pregnancy.

However, despite the absence of clear recommendations for diagnostic GDM thresholds in the early gestational period, the relevance of targeting high-risk mothers is not negligible, as lifestyle modification has been shown to be effective to prevent the later onset of GDM and perinatal complications in these patients [37–40]. This underlines the clinical importance of our study, as we found that glucose concentrations (fasting as well as dynamically assessed) showed good predictive accuracy for both development of GDM and of GDM with a requirement for insulin (i.e. the more severe course of the disease). In another study, we observed, that among several risk markers measured in the first trimester (including patient history and routine biochemical parameters assessed in the fasting state), fasting plasma glucose was the most relevant predictor for progression to GDM [41]. In a Chinese single-centre study, Wu et al recently concluded that an early (although not blinded) OGTT examination performed at a gestational age of 7–14 weeks showed effective risk stratification, which is in line with our findings [42].

Interestingly, the results of our current study indicate that early elevated fasting and OGTT glucose levels were closely associated with insulin resistance and decreased beta cell function, as well as long-term hyperglycaemia, which may further underline their clinical importance for early risk stratification. In this context, it is important to mention that elevated fasting and post-load glucose levels correspond to different pathophysiological features of impaired glucose metabolism [43]: while increased fasting glucose is related to impaired hepatic insulin sensitivity (i.e. the inability of insulin to suppress gluconeogenesis in the liver), elevated postprandial glucose levels (especially 120 min post-load glucose concentrations) rather represent peripheral insulin resistance in the skeletal muscle. Likewise, increased fasting glucose is associated with an early (first phase) defect in beta cell function, whereas increased postprandial glucose reflects a late (second phase) beta cell dysfunction. Consequently, a dynamic test in addition to fasting glucose assessment is required to achieve a complete picture of altered glucose homeostasis. In line with these considerations, multivariable analysis indicated improved predictive accuracy for the combination of fasting and postprandial glucose concentrations, with an AUROC value of 75%. This approach performed quite well compared with previously published risk prediction models based on patient history and fasting laboratory parameters, with AUROC values between 60.7 and 76.9% [41]. Interestingly, early gestational 60 min glucose values remained a strong predictor for the later development of GDM and especially the requirement for insulin even after exclusion of women who met GDM diagnostic criteria and had one or more missing values in early pregnancy. In this regard, previous research has suggested that the 60 min values may reflect metabolic alterations that are not entirely mirrored by fasting and 120 min glucose concentrations [44]. It is also worth mentioning that the 60 min glucose concentration has been shown to be a good predictor for the later development of type 2 diabetes [45–47], including in studies on women with a prior pregnancy complicated by GDM [48].

We observed no association between early gestational glucose values and whether the newborn was LGA, possibly due to the fact that all our patients received appropriate treatment for GDM following diagnosis after 24 weeks of gestation. This is in line with our observation that women who developed GDM in later pregnancy showed comparable pregnancy outcomes to those who remained NGT. In contrast, the recent publication from the TOBOGM cohort suggested that early gestational OGTT glucose concentrations were positively associated with LGA offspring and other perinatal complications [12].

Regarding other investigated biomarkers, we found that lower adiponectin as well as elevated fasting insulin and early gestational triglycerides/triacylglycerols were significantly associated with the later development of GDM. Likewise, these biomarkers (especially fasting insulin and triglycerides/triacylglycerols) showed significant associations with impaired whole-body insulin sensitivity and decreased beta cell function. The association of early gestational dyslipidaemia with disturbed glucose metabolism and later development of GDM was previously assessed in other studies with comparable findings [14, 15, 49, 50]. Noteworthy, a revised triglyceride–glucose index that includes information on fasting triglycerides/triacylglycerols in addition to glucose, insulin and body composition was recently proposed as a promising novel surrogate marker of insulin sensitivity, especially in pregnant women [51]. Moreover, elevated maternal triglycerides/triacylglycerols have been shown to be associated with adverse maternal and fetal outcomes independently of hyperglycaemia or obesity, emphasising the possible relevance of triglycerides/triacylglycerols as a risk marker for mothers who remain NGT during pregnancy [52, 53]. Similarly to maternal lipids, adiponectin plays an important role in the regulation of glucose homeostasis, as it is significantly associated with whole-body insulin sensitivity and normal beta cell function even in the periconceptional period [54, 55]. More specifically, circulating concentrations of adiponectin have been assumed to be indicative of fat tissue health and quality [56]. However, despite the promising properties of adiponectin, our study indicates that its prognostic value for diagnosis of GDM on the basis of the IADPSG criteria is only moderate, in line with previous observations [55]. No notable associations were found for the remaining parameters, including lipocalin-2, PlGF, glycated albumin or glycated fibronectin.

Of note, a detailed analysis of the importance of the various biomarkers suggested that the prognostic value of all investigated biomarkers was inferior compared with fasting and especially dynamically assessed glucose during the OGTT at start of pregnancy. The results remained comparable in two sensitivity analyses after excluding patients who met the WHO criteria and alternative diagnostic criteria in early pregnancy. However, the resulting lower sample sizes should be taken into consideration when interpreting these results. Hence, future research questions need to determine applicable thresholds and suitable interventions for mothers at particularly high risk in early pregnancy to prevent the later development of GDM.

Our approach of a multicentre study in which patients and medical staff were blinded to the test results is a clear advantage, as an impact of the examination at the start of pregnancy on dietary and lifestyle habits can be excluded. The detailed investigation of parameters of glucose metabolism, although performed in a subgroup, is another advantage. As a limitation, it should be mentioned that our study was not designed or powered to evaluate (or validate) possible glucose thresholds in the first trimester.

In summary, we found that an OGTT in early pregnancy can predict the later development of GDM as well as the requirement for insulin with good accuracy. The predictive performance of glucose concentrations was better compared with other biochemical parameters assessed in our study, as well as maternal age and pre-gestational BMI. Therefore, our data support use of the OGTT as an important tool for early gestational risk stratification. Future studies are required to evaluate possible thresholds and suitable interventions targeting mothers at particularly high risk to prevent onset of GDM in later pregnancy.

## Supplementary Information

Below is the link to the electronic supplementary material.

Supplementary file1 (PDF 171 KB)

## Data Availability

Data are available on request from the corresponding author.

## Abbreviations

AUROC: Area under the receiver operating characteristic curve
GDM: Gestational diabetes mellitus
LGA: Large for gestational age
NGT: Normal glucose-tolerant
PlGF: Placental growth factor
SGA: Small for gestational age
