# Supplementary material for: The utility of early gestational OGTT and biomarkers for the development of gestational diabetes mellitus: an international prospective multicentre cohort study
Source: Diabetologia. 2025 Aug 16;68(11):2511–22. doi: 10.1007/s00125-025-06517-0 (PMC12534333; doi:10.1007/s00125-025-06517-0)

ESM Figure 1: Variable importance scores for the prediction of GDM (A) and GDM with need of insulin (B), after exclusion of women who met alternative diagnosis criteria at early pregnancy (fasting: 5.3 mmol/l, and/or 60 min: 10.6 mmol/l, and/or 120 min: 9.0 mmo

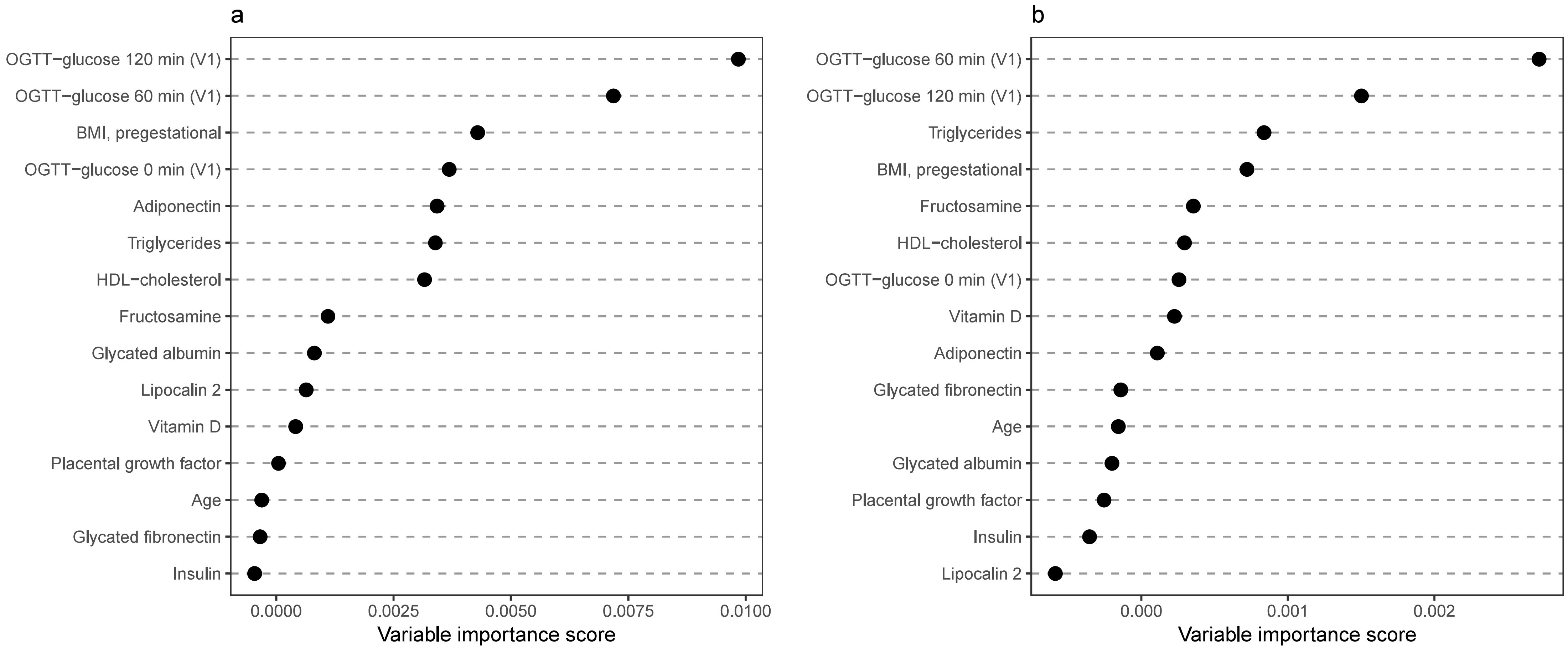

ESM Figure 2: Variable importance scores for the prediction of GDM (A) and GDM with need of insulin (B), after exclusion of women who met the WHO criteria at early pregnancy (fasting: 5.1 mmol/l, and/or 60 min: 10.0 mmol/l, and/or 120 min: 8.5 mmol/l).

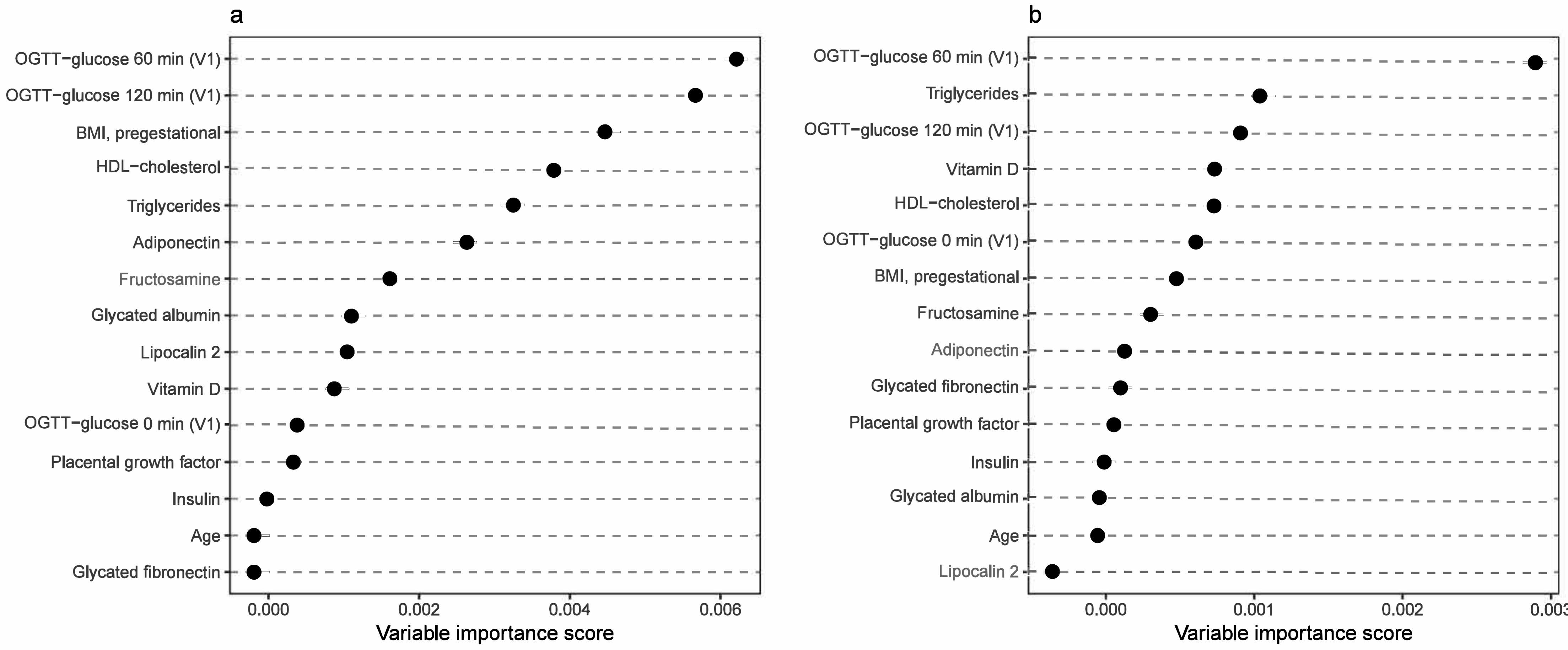

Supplement: Supplementary file 1 — Supplementary file1 (PDF 171 KB) [file 125_2025_6517_MOESM1_ESM.pdf]
